# Genomics of vancomycin-resistant *Enterococcus faecium*

**DOI:** 10.1099/mgen.0.000283

**Published:** 2019-07-22

**Authors:** Claire Gorrie, Charlie Higgs, Glen Carter, Timothy P. Stinear, Benjamin Howden

**Affiliations:** ^1^ Microbiological Diagnostic Unit Public Health Laboratory, Department of Microbiology and Immunology, The University of Melbourne at the Peter Doherty Institute for Infection and Immunity, Melbourne, Australia; ^2^ Department of Microbiology and Immunology, The University of Melbourne at the Peter Doherty Institute for Infection and Immunity, Melbourne, Australia; ^3^ Doherty Applied Microbial Genomics, Department of Microbiology and Immunology, The University of Melbourne at the Peter Doherty Institute for Infection and Immunity, Melbourne, Australia; ^4^ Department of Infectious Diseases, Austin Health, Heidelberg, Australia

**Keywords:** *Enterococcus faecium*, antibiotic resistance, whole-genome sequencing

## Abstract

Vancomycin-resistant *Enterococcus faecium* (VREfm) is a globally significant public health threat and was listed on the World Health Organization’s 2017 list of high-priority pathogens for which new treatments are urgently needed. Treatment options for invasive VREfm infections are very limited, and outcomes are often poor. Whole-genome sequencing is providing important new insights into VREfm evolution, drug resistance and hospital adaptation, and is increasingly being used to track VREfm transmission within hospitals to detect outbreaks and inform infection control practices. This mini-review provides an overview of recent data on the use of genomics to understand and respond to the global problem of VREfm.

**Impact Statement**

The rise of vancomycin-resistant *Enterococcus faecium* (VREfm) poses a public health threat, especially within healthcare settings. To monitor and limit the spread of VREfm we need to understand how it evolves and acquires vancomycin resistance, how transmission networks are operating and how VREfm is developing resistance to last-line antibiotics. This mini-review provides an overview of how whole-genome sequencing is being used to address these questions.

## Introduction

**Fig. 1.**
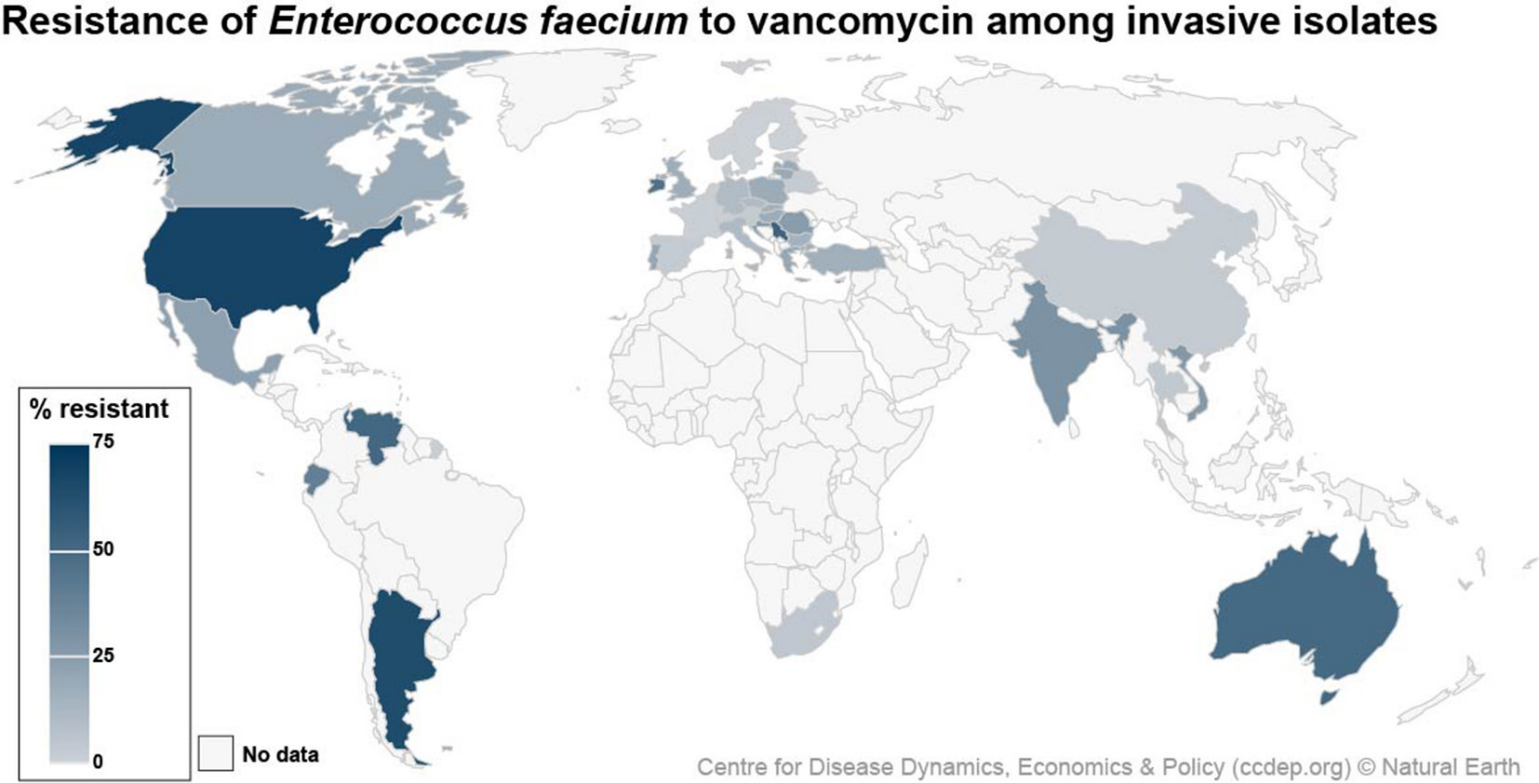
Rates of invasive VREfm worldwide. Map adapted from The Centre for Disease Dynamics, Economics and Policy (CCDEP), Resistance Map: Antibiotic Resistance, accessed 11 January 2019, at https://resistancemap.cddep.org/AntibioticResistance.php. Data include aggregated resistance rates for isolates (includes intermediate resistance) from blood and cerebrospinal fluid (i.e. invasive) from inpatients of all ages. Because of differences in the scope of collections and testing methods, caution should be exercised in comparing across countries.

*Enterococcus faecium *, a species of Gram-positive cocci, is recognized as a globally important opportunistic pathogen that is capable of causing a range of human infections associated with high mortality rates, particularly in hospitalized individuals [1, 2]. Enterococci are inherently resistant to a number of antimicrobial classes, and over recent decades there has been a significant increase in the rates of acquired antimicrobial resistance (AMR) in *E. faecium *, including vancomycin-resistant *E. faecium* (VREfm) [3, 4]. Since the first reports in the late 1980s, VREfm have come to represent a globally significant public health threat, such that in some regions (e.g. the USA and Australia) up to 50 % or more of all blood culture isolates of *E. faecium* are vancomycin-resistant (Fig. 1) [5].

The World Health Organization (WHO) recently published their list of priority bacterial pathogens for which new antibiotics are urgently needed, and VREfm is listed in the high-priority category [6]. As well as being resistant to vancomycin, VREfm usually exhibit resistance to a wide range of other antibiotics, including intrinsic resistance to cephalosporins, lincosamides, aminoglycosides and trimethoprim/sulfamethoxazole, almost universal resistance to penicillins, and increasing reports of acquired resistance to last-line agents such as linezolid and daptomycin [1, 5, 7, 8].

Genomic data can be used to understand the evolution of VREfm, determine resistance mechanisms and investigate the potential sources of human VREfm infection (Table 1). Clones of VREfm associated with invasive human infection are recognized as primarily healthcare-associated pathogens. Approaches to molecular typing of VREfm are therefore important for informing molecular epidemiology and methods of infection prevention in the healthcare setting. Approaches such as multilocus sequence typing (MLST) have been used to classify *E. faecium* [9], but this approach has a number of limitations, including the lack of fine resolution necessary for inferring putative transmission. Phylogenetic analyses based on whole-genome sequencing (WGS) data are increasingly being used in combination with – or in place of – MLST in order to overcome this limitation and identify transmission events [10, 11].

**Table 1.** Selected publications using WGS to investigate genomic features, evolution and transmission of *E. faecium
*

**Category**	**Publication**	**Aims**	**Main findings**
**Complete genomes**	Lam *et al*. [12]	Publish a fully assembled, finished VREfm genome	Published a 3.0 MB complete genome of an ST17 isolate from Australia
Qin *et al*. [13]	Publish a fully assembled, finished Efm genome	Published a complete Efm genome (ST18) and confirmed that hospital-associated and community-associated Efm are found in distinct genomic clades
**Evolution of VRE**	Galloway-Peña *et al*. [14]	Study the diversity of Efm and identify any hospital- or community-associated clades.	Efm can be split into two distinct clades, one hospital-associated and the other community-associated. The differences between the two clades occur at the core genome level and long pre-date the modern antibiotic era
de Been *et al*. [15]	Understand the role of recombination in the core genome of Efm strains	Recombination has a major impact on the Efm genome. The Efm gut commensals are the most important reservoir for donating DNA to hospital-associated Efm
Lebreton *et al*. [16]	Use genomics to understand how Efm emerged as a leading hospital pathogen	Efm is split into two distinct clades (human-associated and animal-associated) and the clade structure parallels changes in urbanization and animal domestication
Raven *et al*. 2016 [17]	Study the genomic variability within the Efm population of the United Kingdom and Ireland	Supports the description of two distinct clades, hospital-associated (A) and community-associated (B), but does not support the subdivision of the hospital-associated clade
Theodore *et al*. [18]	Understand the genetic similarity between Efm isolated from human bloodstream infection samples and livestock/environmental samples	Most strains infecting patients are largely distinct from those from isolated from livestock, with limited sharing of strains and resistance genes
**Hospital/outbreak studies**	Brodrick *et al*. [11]	Use WGS to investigate an Efm outbreak in a hospital network	VRE isolated from patients identified as long-term carriers were closely related to VRE associated with bloodstream infections in a nearby hospital
Carter *et al*. [19]	Use WGS to characterize recently identified VREfm isolates non-typeable by MLST	Non-typeable VREfm isolates were identified that lack one of the genes used in the MLST scheme (*pstS*)
Howden *et al*. [20]	Use genomics to better understand the epidemiology of Efm within a large hospital and investigate the reasons for failure of infection control strategies	VanB VREfm generation within a patient appears common, presumably occurring in the human bowel during antibiotic therapy. Potential implications fort infection control strategies, and the importance of hospital-adapted VSE
Pinholt *et al*. [21]	Investigate the epidemiology and clonal relatedness of VREfm isolates in Danish hospitals in 2012–13 using WGS	WGS typing has the greatest discriminatory power in determining transmission networks. Genomics revealed a polyclonal structure of the VREfm outbreak
Raven *et al*. [22]	Undertake a retrospective genomic based transmission investigation at a single hospital in the United Kingdom	WGS is important for accurate and effective infection control practices and can identify transmission events that are missed by conventional outbreak investigation techniques
Raven *et al*. [23]	Investigate the degree of relatedness in patients with recurrent *E. faecium* bacteraemia and investigate the strain relatedness in patients with apparent co-infection of VREfm and VSEfm	Most reinfections were driven by new strains, often acquired from the hospital. Most patients with mixed VREfm and VSEfm infection had unrelated strains
Pinholt *et al*. [24]	Investigate the transmission events and clonal relatedness of VREfm in Copenhagen, Denmark	VREfm emerged owing to importation of a successful VREfm clone that rapidly transmitted to most hospitals in the region

This mini-review will describe how genomic data have been used to understand the mechanisms of vancomycin resistance in VREfm, the clonal structure and evolution of *E. faecium* and VREfm clinical isolates, and emerging mechanisms of resistance to last-line antibiotics.

### Mechanisms of vancomycin resistance

Vancomycin resistance in hospital-adapted *E. faecium* can be conferred by a number of van operon variants, though the most common, and therefore most clinically relevant, are the VanA and VanB operons (Table 2) [3, 5]. Both van operons encode a ligase that alters the target binding site of vancomycin, which is critical for antibiotic binding [25]. Globally, *vanA* VREfm is most common, while in some regions, including in Australia, *vanB* VREfm has been the major issue. However, this trend is now changing, with a recent rapid increase in *vanA* VREfm being seen in some parts of the Australia [26] and some parts of Europe, such as Germany, reporting a rapid increase in *vanB* VREfm [27]. Other van types are less commonly reported.

**Table 2.** Characteristics of van operons in *Enterococcus* spp.

**Van operon**	**Level of resistance**	**Location and mobility (transposon**)	**References**
Vancomycin	Teicoplanin
*vanA*	High	High	Chromosome, transferable (Tn*1546*), plasmid	[28, 29]
*vanB*	High (variable)	Susceptible	Chromosome, transferable (Tn*1547*, Tn*1549*, Tn*5382*)	[28, 30–33]
*vanC*	Low	Susceptible	Chromosome	[34–37]
*vanD*	Low to high (variable)	Low to high (variable)	Chromosome	[38–40]
*vanE*	Low to moderate	Susceptible	Chromosome	[28, 35, 41]
*vanG*	Low	Susceptible	Chromosome, transferable	[42]
*vanL*	Low	Susceptible	Chromosome	[43]
*vanM*	High	High	Unknown, transferable	[44]
*vanN*	Low	Susceptible	Plasmid, transferable	[45]

Note: table adapted from [8] with additional material as per the references column.

The VanA operon is carried on Tn*1546*-type transposons, which display a high degree of heterogeneity. Point mutations, deletions and various insertion sequences have all been associated with Tn*1546*-type transposons [46, 47]. Tn*1546* is found on pRUM-like plasmids (predominant in the USA) and Inc18 plasmids (predominant in Europe) [5], however, much remains unknown and the molecular epidemiology of VanA-containing plasmids may differ significantly between regions. In addition to contributing to the spread of vancomycin resistance, Inc18 and pRUM-like plasmids can also harbour resistance genes for multiple other antibiotics [48].

The human gastrointestinal tract is a natural reservoir of non-enterococcal species containing the VanB operon [49], and the *in vivo* transfer of a VanB-containing transposon from a non-enterococcal species to vancomycin-susceptible *E. faecium* may explain the emergence of VREfm in some cases [20]. The human bowel has also been shown to be a potential reservoir for the VanD and VanG operons [50]. The natural reservoir of the VanA operon remains unknown [49, 51].

### Classification and adaptative evolution of VRE clones associated with human colonization and infection

#### Strain classification methods and the role of WGS

Historically, VREfm have been classified based on a number of typing methods, including MLST, which characterizes the diversity of isolates based on the allelic variation of seven housekeeping genes in order to assign a sequence type (ST) [9]. Classifying VREfm, and *E. faecium* more broadly, by ST has allowed the identification of persistent or dominant clones on both a local and a global scale; however, MLST has a number of significant limitations, including poor discriminatory power [21]. Additionally, some VREfm isolates have been identified that lack the *pstS* gene, one of the seven MLST loci [19], and recombination has been shown to affect the VREfm genome in the regions of key MLST loci [17, 20]. The highly dynamic nature of the *E. faecium* genome, with the constant flux of accessory genes and horizontal gene transfer events [52, 53], further exacerbates the shortcomings of the MLST method. WGS has proven to be the ultimate molecular typing method for VREfm [21, 54, 55]. Comparative analyses have been based on pairwise single-nucleotide polymorphism (SNP) comparisons, and more recently a core genome MLST (cgMLST) approach using 1423 target genes that has a similar resolution to that of an SNP-based approach [15].

### VREfm clonal types associated with human disease

The majority of sequenced strains are human clinical isolates, with an overrepresentation of isolates from Europe and North America, but these only form a small part of the total *E. faecium* population [52]. A comparison of 100 core genes in all available *E. faecium* genomes initially found that most of the genes split into 2 clades, differing by 3.5–4.2 % nucleotide divergence [14]. The two distinct, highly genetically divergent clades can be grouped by origin: clade A (hospital-associated; consisting mainly of common clinical isolates and some isolates of community origin) and clade B [community associated; consisting almost entirely of isolates from the community (both human and animal)] [14, 16–18]. Initial comparative genome studies suggested that clade A could be subdivided into clade A1 (hospital-associated) and clade A2 (animal-associated) [16, 18], a distinction not fully supported by some later studies [17]. The majority of hospital-associated VREfm are part of clonal complex 17 (CC17) and common STs within CC17 include ST17, ST18, ST80 and ST203 [56].

Genomic studies on global populations of *E. faecium *, including VREfm, have revealed that in some cases close genetic relationships exist between *vanB* VREfm isolates and vancomycin-susceptible *E. faecium* (VSE) from the same institutions, suggesting possible frequent generation of new *vanB* VREfm clones within resident VSE isolates [20], while other studies indicate frequent intra-regional and inter-regional spread of VREfm clones [17, 21, 53].

### Role of genomic plasticity in adaptation to the healthcare environment

WGS has revealed that horizontal gene transfer – involving plasmids, prophages, genomic islands, homologous recombination and the recently identified enterococcus cassette chromosome [57] – is an important driver of the diversity and adaptive evolution of the dynamic *E. faecium* genome [52, 58, 59]. Complete sequencing of VREfm genomes has revealed a large number of these accessory elements, as well as many insertion sequences in clade A VREfm genomes [12]. The ability of *E. faecium* to acquire foreign DNA has been central in the evolution of drug resistance and hospital adaptation in this species. At the core genome level, chromosome regions have been identified that predominantly recombine in specific hospital-associated *E. faecium* strains [59]; these regions include those involved in the biosynthesis of cell wall polysaccharides and carbohydrate uptake and metabolism. Genes involved in these processes have been shown to be important in successful colonization and overgrowth of hospital-adapted *E. faecium* during antibiotic therapy [60], with the gain and loss of carbohydrate metabolism genes reflecting the environmental/colonizing niche of the isolates. Clade B isolates tend to contain genes involved in complex carbohydrate utilization, while clade A, hospital-adapted VREfm, contain genes involved in the utilization of amino sugars found at the epithelial surface [61].

A pan-genome analysis by van Schaik *et al*. [58] indicated that the total available gene pool within the species is expanding (open pan-genome), with *E. faecium* able to acquire genes from many different bacteria. However, the majority of imported DNA is from other *E. faecium* populations [53, 59], with clade B (community-associated) being the most important reservoir for foreign DNA for clade A (hospital-associated) [59]. Multidrug-resistant, hospital-adapted *E. faecium* have also been shown to lack clustered regularly interspaced short palindromic repeats (CRISPR) self-defence systems that protect from genomic modification by plasmids and phage, thereby making them more susceptible to changes through horizontal gene transfer [62]. A recent study by Pinholt *et al*. [24] comparing over 800 VREfm strains suggested that the success of a dominant VREfm clone was due to the acquisition of a heterogenous accessory genome, with no single successful combination of accessory genes. This flexibility and large variation in the accessory genes is an important driver of the evolution of *E. faecium* and its adaptation to the healthcare environment [53, 58, 59], as it is readily able to acquire genes that increase fitness under adverse conditions.

WGS has also shown that the *E. faecium* genome contains a variety of loci encoding key virulence factors that make it particularly suited to persistence in a hospital environment and cause invasive infections, as summarized in a review by Gao *et al*. [63]. A recent study identified a unique botulinum neurotoxin gene cluster (host species target to be determined) in a commensal strain of *E. faecium* and demonstrated the ability of *E. faecium* to horizontally acquire and possibly disseminate this gene cluster [64].

### Role of VREfm genomics in hospital infection control

WGS is providing the capacity for highly discriminatory identification of pathogen outbreaks and transmission, including in the hospital setting. The application of WGS to putative hospital outbreaks of VREfm has been reported, with clear evidence of utility (Table 1), and in some cases has resulted in changes in infection control practices [22, 54, 65]. WGS of VREfm has particular value in identifying complex transmission networks, especially where multiple wards are involved, where patients move wards between VREfm acquisition and the development of a clinical infection, and where environmental contamination is contributing [22]. Although many unknowns remain with regard to percentage genome-wide nucleotide identity and SNP thresholds for identifying transmission events, using WGS data complemented by space–time epidemiological data can allow these more complex transmission links to be defined, informing appropriate interventions. In Australia, WGS has additionally been used to detect and describe the emergence of new clones of VREfm that have become regionally important, including *pstS* null strains (now ST1421-ST1425), which lack one of the MLST typing genes [19, 66].

### Mechanisms and emergence of resistance to last-line antibiotics

Treatment options for VREfm infections, especially *vanA* VREfm, are limited and last-line antibiotics such as linezolid, daptomycin, tigecycline and possibly quinupristin/dalfopristin are often needed. The majority of linezolid-resistant VREfm cases have been found in North America and Europe [67] and although the majority of linezolid-resistant cases appear to emerge after linezolid treatment, linezolid-resistant enterococci have been shown to emerge in the absence of linezolid treatment [68, 69]. Of particular concern are the *optrA, poxtA* and *cfr* resistance genes, which can be rapidly disseminated in *E. faecium* on mobile genetic elements [70–72], particularly under the selective pressures found in clinical settings. Daptomycin non-susceptibility has been shown to emerge in VREfm both during daptomycin therapy [73] as well as in the absence of previous exposure to the antibiotic [74]. The recent review by Ahmed and Baptiste [8] outlines the genotypes associated with resistance to last-resort antibiotics such as these. Teicoplanin remains an option for therapy for some *vanB* VREfm infections, although the emergence of resistance during therapy has been reported, especially in high-bacterial-load infections, and WGS-based studies have shown that this may be due to mutations in the *vanS* region of the VanB operon [75]. Genomics has been an important tool in understanding the mechanisms of resistance to last-line agents, especially given the often polygenic nature of resistance mechanisms.

In addition to growing drug resistance, a recent study has also suggested that strains of VREfm are becoming more tolerant to alcohol-based disinfectants, with isolates collected after 2010 being significantly more tolerant to killing by alcohol compared to older isolates in Australia through mutations occurring in cell wall-associated transport proteins [76]. Other studies have shown that repeated exposure to chlorhexidine, an antiseptic used in infection control practices, can lead to reduced chlorhexidine susceptibility [77, 78]. The wider consequences of these findings on hospital-based infection prevention strategies remain to be seen, but these studies further highlight the highly adaptive nature of VREfm in the face of selective pressures. These data provide a warning about potential future adaptations that will pose further challenges to the control of this important nosocomial pathogen.

### Conclusions and view to the future

VREfm represent a significant public health threat, causing opportunistic invasive infections that are often broadly resistant to available antibiotic treatments. *E. faecium* are highly adaptive and evolve rapidly; the level of variation within a population is rarely accurately represented by traditional typing approaches, although the use of WGS is now providing important new insights into the genetic mechanisms underlying the evolution and adaptation of this nosocomial pathogen. Studies implementing WGS have been able to define both local and large-scale spread of clones, as well as elucidating genomic aspects related to host specificity, resistance and hospital adaptation. These studies have also highlighted the importance of vancomycin-susceptible *E. faecium* (VSE) as a nosocomial pathogen that underpins the further evolution and spread of VREfm. These studies have helped identify risk factors, adaptations and mechanisms for transmission and treatment failure that now need to be leveraged to inform and direct efforts to limit the further evolution and spread of VREfm. While in some regions the burden of VREfm in the healthcare setting may appear overwhelming, the precision of WGS has the potential to reveal the complexity of this problem and provide the evidence for real action.
